# RAGE and AGEs in Mild Cognitive Impairment of Diabetic Patients: A Cross-Sectional Study

**DOI:** 10.1371/journal.pone.0145521

**Published:** 2016-01-08

**Authors:** Pin Wang, Rong Huang, Sen Lu, Wenqing Xia, Rongrong Cai, Haixia Sun, Shaohua Wang

**Affiliations:** 1 Department of Endocrinology, Affiliated ZhongDa Hospital of Southeast University, Nanjing, PR China; 2 Department of the Intensive Care Unit, Sichuan Academy of Medical Science & Sichuan Provincial People's Hospital, Chengdu, Sichuan Province, PR China; University of Pittsburgh, UNITED STATES

## Abstract

**Objective:**

Receptor for advanced glycation end products (AGEs; RAGE) binds to both AGEs and amyloid-beta peptides. RAGE is involved in chronic complications of type 2 diabetes and Alzheimer’s disease. We aimed to investigate the roles of RAGE, AGEs and the Gly82Ser polymorphism of RAGE in mild cognitive impairment (MCI) among type 2 diabetes patients.

**Methods:**

Of the 167 hospitalized type 2 diabetes patients recruited, 82 satisfied the diagnostic criteria for MCI, and 85 matched control individuals were classified as non-MCI. Demographic data were collected, and the soluble RAGE (sRAGE) concentrations, serum AGE-peptide (AGE-P) levels, RAGE Gly82Ser genotype and neuropsychological test results were examined.

**Results:**

The MCI group exhibited a decreased sRAGE level (0.87±0.35 vs. 1.05±0.52 ng/ml, *p*<0.01) and an increased serum AGE-P level (3.54±1.27 vs. 2.71±1.18 U/ml, *p<*0.01) compared with the control group. Logistic regression analysis indicated that each unit reduction in the sRAGE concentration increased the MCI risk by 54% (OR 0.46[95% CI 0.22–0.96], *p* = 0.04) and that each unit increase in the AGE-P level increased the MCI risk by 72% in the type 2 diabetes patients (OR 1.72[95% CI 1.31–2.28], *p<*0.01). The serum sRAGE level was negatively correlated with the score on the trail making test-B (TMT-B) (r = -0.344, *p* = 0.002), which indicates early cognitive deficits related to diabetes. Moreover, the AGE-P level was positively correlated with multiple cognitive domains (all *p*<0.05). No significant differences in the neuropsychological test results or serum RAGE concentrations between the different RAGE genotypes or in the RAGE genotype frequencies between the MCI and control groups were identified (all p>0.05).

**Conclusions:**

The RAGE pathway partially mediates AGE-induced MCI in diabetic patients. The serum AGE-P level may serve as a serum biomarker of MCI in these individuals, and sRAGE represents a predictor and even a potential intervention target of early cognitive decline in type 2 diabetes patients.

**Trial registration:**

Advanced Glycation End Products Induced Cognitive Impairment in Diabetes: BDNF Signal Meditated Hippocampal Neurogenesis ChiCTR-OCC-15006060

## Introduction

Diabetes mellitus affects the brain and other organs, such as the kidneys and eyes, as well as the peripheral nervous system. It has been demonstrated that individuals with diabetes have a much higher (up to a two-fold greater) risk of Alzheimer’s disease [1, 2]. Individuals with type 2 diabetes are at a 50 to 60% increased risk of developing mild cognitive impairment (MCI), which has an annual rate of progression to dementia of 15% [3, 4]. Type 2 diabetes contributes to cognitive impairment via several associated biological conditions, including hyperglycemia, insulin deficiency, glucose-mediated toxicity, and amyloid-beta (Aβ) peptide accumulation, although the underlying pathogenesis requires further study [5].

Advanced glycation end products (AGEs) are a complex and heterogeneous group of compounds that have been implicated in diabetes-related complications. CML and pentosidine, two AGEs that have been shown to contribute to Alzheimer's disease pathology and vascular dementia, have predominantly been isolated from CSF, and a few simple and rapid analytical procedures are available for detecting these molecules. The evaluation of AGE-peptides is a valuable and useful alternative to the measurement of AGEs in the bloodstream; of AGE-peptides for AGE-peptide evaluation are lacking [6, 7]. AGE-P is released from naturally degraded AGEs in tissue and binds to a small peptide (AGE-peptide) of less than 10 kDa [8], which acts as a reactive intermediate. The levels of AGE—P can be determined by immunochemical methods [9] such as flow injection assay; such methods were found to be superior to enzyme-linked immunosorbent assay (ELISA) and fluorescence spectroscopy for the examination of AGEs [10]. Furthermore, AGE-P exhibits the same toxic activity as AGEs, and AGE-P has been recognized to be responsible for complications of diabetes such as diabetic nephropathy [11, 12]. More importantly, a reliable and reproducible assay for determining the serum AGE-P concentrations has been successfully validated in previous studies [13–15].

Receptor for AGEs (RAGE) mediates most of the toxic effects of AGEs that are associated with diabetic complications. Additionally, RAGE functions as a signal-transducing cell surface receptor of Aβ peptides and promotes Aβ peptide accumulation and deposition in amyloid plaques, which accumulate during Alzheimer’s disease development [16, 17]. However, whether RAGE is involved in diabetes-related dementia is unknown.

In addition to the full-length membrane-bound isoform of RAGE (mRAGE), which is expressed on the cell surface, soluble RAGE (sRAGE), which circulates in serum, is produced via either protease-mediated cleavage of mRAGE (cRAGE) or alternative splicing of the RAGE gene (esRAGE) [18, 19]. As it lacks a transmembrane domain and circulates in plasma, sRAGE contributes to the removal or neutralization of circulating ligands and functions as a decoy by competing with mRAGE for ligands [20]. The plasma sRAGE level has been associated with vascular complications, and sRAGE has recently been proposed as a biological indicator of cognitive decline [21–23].

The RAGE gene is located on chromosome 6p21.3, and a polymorphism at codon 82 (GGC→AGC) of RAGE causes an amino acid substitution from glycine to serine within its ligand-binding domain [24]. Cells that express this S82 isoform of RAGE exhibit enhanced ligand-binding affinity, increased inflammatory mediator generation, and a decreased plasma sRAGE concentration [25, 26]. Many studies have demonstrated that the Gly82Ser polymorphism is independently involved in cardiovascular diseases, diabetes complications, and dementia, particularly Alzheimer’s disease [27, 28]. However, no studies of the association of RAGE Gly82Ser with diabetes-related MCI have been reported.

This cross-sectional study aimed to investigate the roles of RAGE, AGEs and the RAGE Gly82Ser polymorphism in type 2 diabetes-associated MCI, especially in different cognitive domains. Our results will facilitate the prediction and early intervention of diabetes-associated MCI.

## Research Design and Methods

### 2.1. Clinical subjects and study design

This study was conducted at the Endocrinology Division of the ZhongDa Hospital of Southeast University. All individuals provided written informed consent prior to participation in the study, which was approved by the Research Ethics Committee of the Affiliated ZhongDa Hospital of Southeast University (S2 File).

**Fig 1:**
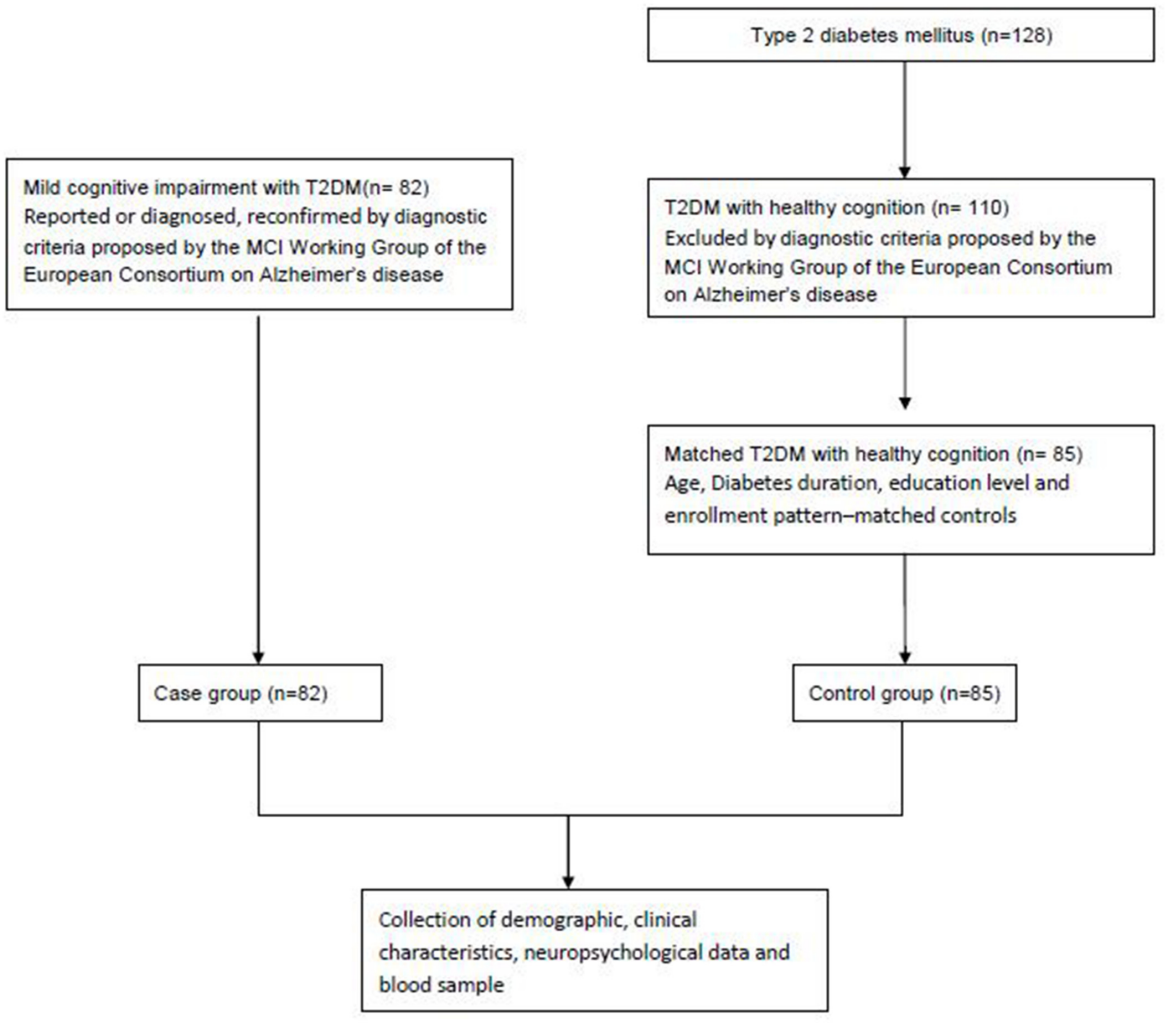
Flow chart of study population.

This case-control study recruited 167 (65 female and 102 male) hospitalized patients who satisfied the diagnostic criteria of type 2 diabetes [29]. All participants were right-handed Han Chinese individuals who had at least eight years of education. Eighty-two patients (33 females, 49 males, mean±SD age = 60.15±7.47 years) satisfied the following diagnostic criteria for MCI proposed by the MCI Working Group of the European Consortium on Alzheimer’s Disease [30]: 1) cognitive complaints from the patient or the patient’s family; 2) a reported decline in cognitive functioning relative to that in the past year by the patient or the patient’s guardian (CDR score of 0.5); 3) cognitive disorder as evidenced by a clinical evaluation (impairment in memory or some other cognitive domain); 4) absence of major limitations in activities of daily living (ADL score≥26); and 5) absence of dementia (based on the DSM-IV criteria). Eight-five hospitalized type 2 diabetes patients (32 females, 53 males, mean±SD age = 59.67±7.79 years) with healthy cognition who were matched to the type 2 diabetes patients diagnosed with MCI according to age, sex, education level, and diabetes duration were also selected. Individuals with a prior history of known stroke, head injury, alcoholism, Parkinson’s disease, epilepsy, major depression or any other neurological or psychiatric illness, or any major medical illness (e.g., cancer, anemia, or thyroid dysfunction) and those who had recently used a medication that might interfere with cognitive testing (including central B-blockers; anti-Parkinson's disease drugs; nerve sedatives; narcotic analgesics; benzodiazepines; barbiturates; short-acting anxiolytic or sedative drugs used more than twice a week; drugs with significant cholinergic or anticholinergic side effects; antiepileptic drugs; warfarin; donepezil hydrochloride, memantine hydrochloride; or any drug recently approved for the treatment of AD based on clinical trials) were excluded from this study (Fig 1).

### 2.2. Clinical measurements

#### 2.2.1 Clinical data collection

Demographic characteristics, including age, gender, educational level/number of years in school, ethnicity, and occupation, were collected. The participants’ medical histories (the duration of diabetes was calculated from the time of disease diagnosis by a physician), drug use, and physical measurements (including blood pressure, weight, height, and waist circumference) were also collected using standardized methods. Blood and urine samples were obtained to determine the fasting blood glucose, HbA1c, triglyceride, total cholesterol, low density lipoprotein cholesterol (LDL-C), high density lipoprotein cholesterol (HDL-C), creatinine, uric acid, free triiodothyronine, free thyroxine, thyroid stimulating hormone and c peptide levels after undergoing a standard oral glucose tolerance test. Ultrasound was performed to detect liver and carotid artery plaques. The Laboratory Center of the ZhongDa Hospital of Southeast University implements internal and external quality control procedures as directed by the Chinese Laboratory of Quality Control.

#### 2.2.2 Neuropsychological test data

The following neuropsychological tests were administered to evaluate the participants’ verbal and visual functions, semantic memory, attention, psychomotor speed, executive function, and visuospatial skills: the Montreal cognitive assessment (MoCA); the digit span test (DST); the trail making test-A and B (TMT-A and B, respectively); the clock drawing test (CDT); the similarities test (ST); the verbal fluency test (VFT); and the Clinical Dementia Rating (CDR). The MoCA assesses multiple cognitive domains, including attention and concentration, executive functions, memory, language ability, visuoconstructional skills, conceptual thinking, calculation performance, and orientation. The DST assesses both attention and short-term memory. The VFT, in which the ability to rapidly speak is assessed, is very effective in measuring executive functioning and language ability. The CDT is highly valuable for evaluating general cognitive and adaptive functions, such as memory, information processing and vision. The ST measures verbal comprehension. The TMT, which measures attention, visual screening ability and processing speed, is a useful measure of overall cognitive functioning, especially executive function. The CDR was used to exclude patients with dementia. These tests were performed by an experienced neuropsychiatrist who was blinded to the study design. None of the participants exhibited audiovisual or motor coordination deficits that could have affected test performance.

### 2.3. Serum levels of sRAGE and AGE-P

Blood samples (2 ml) were collected from the diabetic patients and control subjects into tubes that contained EDTA and were then centrifuged at 100×*g* for 15 min. The serum fraction was collected and stored frozen at –80°C. The sRAGE concentration was assessed using Quantikine sandwich ELISA kits (R&D Systems, Minneapolis, MN, USA) according to the manufacturer’s instructions.

The AGE-P level was determined using a flow injection assay according to the method previously described by Sun [23]. Twenty-microliter serum samples were mixed with 480 μl of trichloroacetic acid (TCA, 0.15 mol/l) and 100 μl of chloroform in micro-centrifuge tubes, which were vigorously shaken and subsequently centrifuged at 13,000 g for 10 min. Twenty microliters of the aqueous layer was injected into the sample injector for high performance liquid chromatography (HPLC, Shimadzu, Japan). The HPLC flow rate was set to 0.5 ml/min, and the emission and excitation wavelengths of the spectrofluorometric detector were 440 and 370 nm, respectively. The samples were analyzed in duplicate, and the peak height mode was used for signal measurement. Standard AGE-Ps (0.1, 0.5, 1, 5, 10, 50, and 100 mg/l) were used to prepare a calibration curve with units of U/ml; 1 U/ml was equal to the concentration of the standard AGE-P obtained from the hydrolysis of 1.0 mg/l AGE from bovine serum albumin (BSA).

### 2.4. Genotyping of the RAGE Gly82Ser polymorphism

Genomic DNA was extracted from the EDTA-treated venous blood samples using a DNA purification kit (Puregene, Gentra Systems, Minneapolis, MN, USA). Polymorphism-based genotyping according to the polymerase chain reaction (PCR)-generated restriction fragment length was performed to detect variants of the RAGE gene (the Gly82Ser polymorphism). The following sense and antisense primers were used: 5′-GTAAGCGGGGCTCCTGTTGCA-3′ and 5′-GGCCAAGGCTGGGGTTGAAGG-3′, respectively. PCR was conducted in a 20 μl reaction mixture containing 1.625 mmol/l MgCl_2_, 0.14 mmol/l deoxynucleotide triphosphates, 1 unit of Taq polymerase, 2 μl of 10× PCR buffer, 200 ng of genomic DNA, and 0.25 μmol/l of each primer. Amplification was initiated at 95°C for 5 min, followed by 35 cycles of denaturation at 94°C for 30 s, 62°C for 45 s, and 72°C for 60 s and a final extension step at 72°C for 10 min. The 397 bp PCR products were digested using 5 U of the restriction enzyme AluI at 37°C for 16 h, followed by electrophoresis on a 2% agarose gel at 75 V for 60 min. Bands corresponding to 249, 123, and 26 bp indicated the presence of the wild-type 82G allele, and those corresponding to 181, 123, 67, and 26 bp indicated the presence of the variant 82S allele.

### 2.5. Statistical analysis

Statistical analyses were conducted using SPSS version 19.0 (SPSS Inc., Chicago, IL, USA). All tests were two-sided, and statistical significance was defined as *p*<0.05. Student’s *t* test and ANOVA were employed for normally distributed variables, and the nonparametric Mann-Whitney *U* and Kruskal-Wallis tests were used for asymmetrically distributed variables. The Chi-squared test was used to test for Hardy-Weinberg equilibrium of the allelic and genotypic distributions (Santiago Rodriguez, Tom R. Gaunt, and Ian N. M. Day, Hardy-Weinberg Equilibrium Testing of Biological Ascertainment for Mendelian Randomization Studies). The reference group comprised the carriers of the wild-type 82Gly/Gly genotype. Pearson or Spearman rank correlation analysis and logistic regression analysis were performed to explore the relationships between the cognitive measures and demographic characteristics, the RAGE Gly82Ser genotype, and the serum sRAGE and AGE-P levels in the MCI group. The cutoff value used in this study for suggested MCI was a MoCA score≤26, with a one-point adjustment of the total score for subjects with fewer than 12 years of education.

## Results

### 3.1. Demographic, clinical and neuropsychological data

The demographic, clinical, and neuropsychological test data for the participants are summarized in Table 1 (Table 1). The MCI and non-MCI patients were well matched in terms of age, sex, education level, and diabetes duration. No significant differences were identified regarding BMI, blood pressure, HbA1c level, blood lipid levels or the percentage of insulin use (all *p*>0.05). Compared with the control group, the diabetic patients with MCI displayed a substantially increased frequency of carotid plaques, coronary heart disease and diabetic nephropathy. Compared with the non-MCI group, the MCI group displayed significantly lower sRAGE concentrations (1.05±0.52 vs. 0.87±0.35 ng/ml, *p<*0.01) and significantly higher serum AGE-P (2.71±1.18 vs. 3.54±1.27 U/ml, *p<*0.01) and blood uric acid levels (254.45 ± 71.04 vs. 289.91 ± 90.63 μmol/L, *p<*0.01). The neuropsychological test scores for multiple cognitive domains were significantly lower in the MCI group than in the non-MCI group (all *p*<0.01).

**Table 1:** Demographic, clinical and cognitive characteristics.

Characteristic	MCI (N = 82)	Non-MCI (N = 85)
**Age (years)**	60.15 ± 7.47	59.67 ± 7.79†
**Female, n (%)**	33(40.2%)	32(37.6%)§
**Education level (years)**	9.28 ± 3.63	10.21 ± 3.95 †
**Smoking, n (%)**	38(46.3%)	33(38.8%) §
**BMI (kg/m**^**2**^**)**	25.06 ± 3.71	25.10 ± 3.10 †
**Hypertension, n (%)**	42(51.2%)	42(49.4%) §
**Diabetes duration (years)**	8.56 ± 5.90	7.65 ± 3.66 †
**HbA1c (%)**	8.83 ± 2.10	8.84 ± 2.00 †
**Fasting blood glucose (mmol/L)**	9.05 ± 2.35	8.78 ± 3.92 †
**Fasting C peptide (ng/mL)**	1.89 ± 1.1	2.09 ± 1.10 †
**C peptide (60 mins)**	2.94 ± 1.51	3.62 ± 1.74 †
**C peptide (120 mins)**	4.60 ± 2.03	5.79 ± 2.93 †
**Triglyceride (mmol/L)**	1.47(0.97–2.18)	1.59(1.19–2.59)‡
**Total cholesterol (mmol/L)**	5.19 ± 1.59	4.89 ± 1.15 †
**LDL cholesterol (mmol/L)**	3.06 ± 0.90	2.95 ± 0.70 †
**HDL cholesterol (mmol/L)**	1.24 ± 0.29	1.27 ± 0.28 †
**Serum creatinine (μmol/L)**	64.61 ± 18.7	65.26 ± 18.98 †
**Blood uric acid (μmol/L)**	289.91 ± 90.63	254.45 ± 71.04 †*
**Free triiodothyronine (pg/mL)**	2.66 ± 0.32	2.74 ± 0.33 †
**Free thyroxine (ng/dL)**	1.23 ± 0.18	1.26 ± 0.17 †
**Thyroid-stimulating hormone (uul/mL)**	2.11(1.27–3.01)	2.23(1.33–3.71) ‡
**Systolic pressure (mmHg)**	130(120–160)	136(126–150) ‡
**Diastolic pressure (mmHg)**	80(78–90)	85(80–90) ‡
**NAFLD**	50(61.0%)	45(52.9%) §
**Carotid plaques**	49(59.8%)	37(43.5%) §*
**Coronary heart disease**	18(21.9%)	9(10.6%) §
**Diabetic nephropathy**	17(20.7%)	8(10.4%)§*
**Drinking**	18(28.1%)	16(25.8%)§
**sRAGE (ng/mL)**	0.87 ± 0.35	1.05 ± 0.52 †*
**AGEs (U/mL)**	3.54 ± 1.27	2.71 ± 1.18 †*
**Use of insulin (%)**	56 (68.3%)	57 (67.1%) §
**Cognition test scores**		
**MoCA**	22 (19–23.3)	27 (26–27) ‡*
**TMT-A**	79.21 ± 26.54	65.28 ± 20.05 †*
**TMT-B**	195.36 ± 64.87	163.4 ± 58.0 †*
**CDT**	4 (3–4)	4(3.5–4) ‡*
**DST**	11 (9–12)	12 (11–13) ‡*
**VFT**	16.43 ± 3.94	19.29 ± 4.36 †*
**ST**	7.54 ± 2.54	9.75 ± 2.69 †*

The data are presented as n (%), the mean±SD, or the median (interquartile range) unless otherwise specified. Abbreviations: BMI, body mass index; MoCA, Montreal Cognitive Assessment; TMT-A and TMT-B, Trail Making Test-A and Trail Making Test-B, respectively; CDT, Clock Drawing Test; DST, Digit Span Test; ST, Similarities Test; VFT, Verbal Fluency Test. MCI, participants with mild cognitive impairment; Non-MCI, participations without MCI; NAFLD, nonalcoholic fatty liver disease; sRAGE, the soluble form of the receptor for advanced glycation end products.

^**†**^Student’s t test was employed for normally distributed variables.

^**‡**^The Mann-Whitney U test was employed for asymmetrically distributed variables.

^**§**^The Chi-square test was employed for categorical variables.

*p<0.05

### 3.2. Relationships between the serum sRAGE and AGE-P concentrations, clinical characteristics, and neuropsychological test scores

A logistic regression model was established, and the results indicated that each unit increase in the sRAGE level was associated with a 54% reduction in disease risk (OR 0.46[95% CI 0.22–0.96], *p* = 0.04), whereas each unit increase in the serum AGE-P level was associated with a 72% increase in MCI risk (OR 1.72[95% CI 1.31–2.28], *p<*0.01) after adjusting for additional risk factors such as the presence of carotid plaques, coronary heart disease or diabetic nephropathy [31–33], which showed significance differences between the two groups and exerted an impact on cognition in type 2 diabetes mellitus patients.

We subsequently explored the associations between the different neuropsychological test scores and the sRAGE and AGE-P levels in the MCI group using Pearson correlation or Spearman rank correlation analyses (Table 2). The sRAGE level negatively correlated with the TMT-B score (r = -0.344, *p* = 0.002). Additionally, significant negative correlations were identified between the serum AGE-P level and the MoCA, DST, VFT, and CDT scores (r = -0.279, -0.443, -0.528, and -0.225, respectively, all *p*<0.05), and the AGE-P levels positively correlated with the TMT-A and TMT-B scores (r = 0.377 and 0.223, respectively, both *p*<0.05).

**Table 2:** Relationships between the serum sRAGE and AGE-P concentrations and the neuropsychological test results.

	Serum AGE-P level		Serum RAGE level	
	r	*p*	r	*p*
**MoCA**	-0.279	0.011*	-0.032	0.774
**TMT-A**	0.377	<0.001*	-0.087	0.435
**TMT-B**	0.223	0.046*	-0.344	0.002*
**CDT**	-0.225	0.042*	0.160	0.151
**DST**	-0.443	<0.001*	0.109	0.328
**VFT**	-0.528	<0.001*	0.114	0.359
**ST**	-0.053	0.672	-0.143	0.247

Abbreviations: MoCA, Montreal Cognitive Assessment; TMT-A and TMT-B, Trail Making Test-A and Trail Making Test-B, respectively; CDT, Clock Drawing Test; DST, Digit Span Test; ST, Similarities Test; VFT, Verbal Fluency Test; sRAGE, the soluble form of the receptor for advanced glycation end products.

*p<0.05

### 3.3 Comparisons of the neuropsychological test scores and the serum RAGE concentrations between different RAGE genotypes and of the RAGE genotype frequencies between the MCI and non-MCI groups

The distributions of the RAGE genotypes were consistent with Hardy-Weinberg equilibrium in both the non-MCI (*χ*^*2*^ = 0.17, df = 1, *p*>0.05) and MCI groups (*χ*^*2*^ = 4.68, df = 1, *p*>0.05) [34]. No significant differences in the RAGE genotype and allele distributions were identified between the MCI and non-MCI groups (*χ*^*2*^ = 2.199, *p* = 0.333, and *χ*^*2*^ = 0.480, *p* = 0.489, respectively, Table 3). Considering the 82Gly/Gly genotype as a reference, the OR of either variant genotype (82Gly/Ser or 82Ser/Ser) was 1.51 (95%CI 0.78 to 2.95, *p* = 0.225) after adjustment for age, education level, sex, hypertension, coronary heart disease, and diabetic nephropathy.

**Table 3:** Distributions of the RAGE genotypes and risk estimates for type 2 diabetes mellitus patients with MCI or normal cognition.

RAGE genotype	MCI, n (%)	Non-MCI, n (%)	Crude OR (95% CI)	p-value	Adjusted OR (95% CI)*	*p*-value
**Overall**	82	85				
**Gly**^**82**^ **allele**	112(68.29)	122 (71.76)				
**Ser**^**82**^ **allele**	52(31.71)	48(28.24)				
**GG**	34(41.46)	43(50.59)	1.000			
**GS**	44(53.66)	36(42.35)	1.55(0.82–2.90)	0.174	1.64(0.83–3.23)	0.157
**SS**	4(4.88)	6(7.06)	0.84(0.22–3.23)**†**	1.0	0.77(0.14–4.29)	0.765
**GS+SS**	48(58.54)	42(59.41)	1.45(0.78–2.66)	0.237	1.51(0.78–2.95)	0.225

The genotype and allele frequencies were compared between the groups using Pearson`s χ^2^ tests, except for the cases labeled with †, in which the frequency was determined using continuity-corrected Pearson`s χ^2^ tests.

Abbreviations: MCI, mild cognitive impairment; sRAGE, the soluble form of the receptor for advanced glycation end products; G, Gly82 allele; S, Ser82 allele.

*Adjusted for age, education level, sex, hypertension, coronary heart disease, and diabetic nephropathy.

The serum RAGE concentration was significantly lower in the MCI group, especially in the 82Gly/Ser subgroup (*p* = 0.003, Table 4), than in the non-MCI group. No difference was identified between the MCI and non-MCI groups in the frequency of the 82Ser/Ser homozygote subgroups or various RAGE genotypes in the case or control subgroup or the total group (all *p*>0.05).

**Table 4:** The serum level of sRAGE (ng/ml) in type 2 diabetes mellitus patients with MCI or normal cognition.

	Genotype			
Group	GG	GS	SS	*p*-value‡
**Non-MCI**	1.04 ± 0.55	1.10 ± 0.51	0.85 ± 0.43	0.557
**MCI**	0.91 ± 0.43	0.80 ± 0.27	1.20 ± 0.35	0.086
***p*-value**†	0.244	0.003	0.254	

The data were expressed as the mean±SD. Abbreviations: MCI: mild cognitive impairment; sRAGE: the soluble form of the receptor for advanced glycation end products; G: Gly82 allele; S: Ser82 allele.

^†^Student’s t test for the comparison of the serum level of sRAGE between Non-MCI and MCI patients with type 2 diabetes mellitus

^‡^One-way ANOVA for the comparison of the serum level of sRAGE between the different genotypes

No differences in the neuropsychological test scores were identified between the RAGE Gly82Ser genotype and the other examined genotypes (all p>0.05, Table 5).

**Table 5:** Comparison of cognitive test scores in the MCI group according to the RAGE genotype.

Cognitive test	GG	GS	SS†	*p*-value‡
**MoCA**	21.12±2.847	21.45±2.72	20.5±3.87	0.747
**TMT-A**	78.09±29.65	78.84±24.64	92.75±19.7	0.580
**TMT-B**	190.41±72.2	193.67±57.8	255.5±54.6	0.161
**CDT**	4(3–4)	4(3–4)	3(3–3.75)	0.614
**DST**	10.53±2.12	10.59±1.60	10.25±1.71	0.936
**VFT**	16.28±4.20	16.63±3.89	16.00±1.73	0.925
**ST**	7.59±2.56	7.59±2.49	6.33±3.51	0.708

The data are presented as the mean±SD or median (interquartile range), unless otherwise specified.

p-value for the difference between the GG, GS, and SS groups. Abbreviations: MoCA, Montreal Cognitive Assessment; TMT-A, Trail Making Test-A; TMT-B, Trail Making Test-B; CDT, Clock Drawing Test; DST, Digit Span Test; VFT, Verbal Fluency Test; ST, Similarities Test; MCI: mild cognitive impairment.

^†^One-way ANOVA.

^‡^Kruskal-Wallis test.

## Discussion

Our results demonstrated that the diabetic patients with MCI displayed a decreased serum RAGE concentration compared with the diabetic patients without MCI. Furthermore, the extent of the decrease in the serum RAGE concentration was correlated with reduced executive function. An elevated serum AGE-P level was associated with a higher MCI risk in the type 2 diabetes patients and was inversely correlated with overall cognitive function in these individuals. No significant differences in the neuropsychological test scores or the serum RAGE concentrations among the different RAGE genotypes or in the RAGE genotype frequencies between the MCI and non-MCI groups were identified.

The included type 2 diabetes patients with MCI exhibited overall cognitive deficits, and many of the clinical parameters of the two groups were matched, including age, sex, education level, and diabetes duration. We discovered that the MCI group exhibited a substantially increased prevalence of carotid plaques, coronary heart disease and diabetic nephropathy compared with the non-MCI group; these findings are consistent with those of a previous report [31–33], as diabetes mellitus is a systemic disease. Both microvascular and macrovascular dysfunction can contribute to changes in cerebral blood flow [35] and can even cause cortical and subcortical atrophy, which influence cognition. Moreover, the accumulation of AGE-P and uric acid was observed in the MCI group. Both AGE-P and uric acid are peripheral markers of oxidative stress [36], and their accumulation suggests an interaction of oxidative stress with the formation of AGEs during the onset of cognitive decline in type 2 diabetes patients.

AGEs contribute to cognitive dysfunction via the AGE-RAGE pathway or via direct toxic effects, such as the release of reactive oxygen species [37, 38] and the reduction of glucose consumption, ATP production and mitochondrial activity in neurons [39]. We determined that each unit increase in the AGE-P level increased the MCI risk by 72% and that each unit increase in the sRAGE level reduced the MCI risk by 54%. Furthermore, a modest association was identified between the sRAGE level and the TMT-B scores but not the MoCA scores. In contrast, a previous cross-sectional study has demonstrated a close relationship between the RAGE levels and MoCA scores [40]. Differences observed between studies may be explained as follows: First, the patients enrolled in both studies were recruited from hospitals, but Chen’s study was designed as a normal cross-sectional study, whereas our study is a case-control study. Thus, the test groups between studies are not equal, and associations between serum sRAGE, AGE-P concentrations, clinical characteristics, and neuropsychological test scores can only be analyzed in the MCI group and not in all of the patients. Second, our patients were relatively younger (60.15 ± 7.47 years; 59.67 ± 7.79 years) than those in Chen’s work (63.90 ± 8.73 years; 62.84 ± 7.94 years). Another recent study showed that RAGE levels in the blood stream increased with MCI progression in diabetic patients [41], their design and the ethnics of the participants were different from that of the current study, additionally, their average ages (73.6 ± 4.8) are ten years older than ours. We found that AGE levels were higher in MCI patients than in healthy-cognition patients and that RAGE level was negatively correlated with MoCA scores. These findings indicate that RAGE levels are expressed more in the elderly and that RAGE may be as compensatory elevation as, AGEs aggregated among the older people, further study is need to clarify the uncertain and difference between us. Unlike other works, our study measured total RAGE levels in serum and found a relationship between decreases in serum RAGE levels and TMT-B scores. In diabetic patients, TMT-B scores are used to evaluate executive function, which is impaired early on in the evolution of diabetes, even in individuals with normal cognition. Executive dysfunction is considered to be an important feature of diabetic cognitive impairment. The results suggested that sRAGE plays a protective role in early cognitive impairment in diabetic patients partly by blocking AGEs-RAGE interactions. At this point, however, we cannot exclude the possibility of counteractive toxic effects arising from Aβ and RAGE interactions.

Moreover, our study demonstrated that each unit increase in the AGE-P level was associated with a 72% increase in MCI risk and that the AGE-P level correlated with the MoCA, DST, VFT, CDT, TMT-A, and TMT-B scores, especially between the AGE-P level and the DST and VFT scores. These findings indicate a relationship between the AGE-P level and attention, concentration, and proceeding speed but not verbal comprehension, as assessed based on the ST scores. As the typical pathological characteristic of diabetes mellitus [42], increased AGE formation represents accumulation of reactive oxygen species [43] and impairment of antioxidant systems. AGEs are involved in the diabetes-related microvascular complications such as diabetic retinopathy [44], diabetic nephropathy [45], and even diabetic neuropathy [46] as well as diabetes-related macrovascular complications [47] by amplifying vascular stress and accelerating atherosclerosis and neointimal expansion [48]. Furthermore, in addition to vascular injury, AGEs participate in the formation and aggregation of Aβ peptides [49] and neurofibrillary tangles (NFTs) [50], which cause neuronal injury in the brain. Consistent with these pathophysiological mechanisms, relationships were observed between the AGE-P level and the MoCA, DST, VFT, CDT, TMT-A, and TMT-B scores, which demonstrated overall cognitive impairment, especially in terms of attention, short-term memory (DST), executive function and language ability (VFT). The higher the AGE-P level, the poorer the cognitive function of the patient. However, little relationship was found between the AGE-P level and the cognitive function of verbal comprehension in our trial. Although impaired verbal comprehension was observed in our MCI group and was even reported in type 1 diabetes patients [51], AGE-P does not appear to serve as a predictor of verbal comprehension impairment in type 2 diabetes mellitus patients. This finding may have occurred because unlike the hippocampus, which is vulnerable to hyperglycemia and inflammation [52], the language and verbal comprehension centers are located in multiple lobes of the brain [53]; thus, multiple factors besides AGE-P may be involved in the impairment of these centers. Nevertheless, AGE-P may serve as a serum biomarker of MCI among diabetic patients.

Previous studies have demonstrated that the Gly82Ser polymorphism of the RAGE gene plays a role in the development of microvascular complications in type 2 diabetes and Alzheimer’s disease [26, 28]. However, we failed to identify a significant association between this gene polymorphism and cognitive decline in type 2 diabetes patients. There are several factors that may explain the negative findings of our study. First, diabetes mellitus is a very complicated disease that can be influenced by several environmental factors and gene mutations. It is difficult to identify a small association in such a varying condition. Second, because the heritability of a disease may originate from many genes, a large sample size is needed to detect a weak effect of a single gene because each gene exerts a very small effect. Third, other gene polymorphisms near the RAGE gene may be involved in the pathogenesis of MCI in type 2 diabetes mellitus patients, and the Gly82Ser polymorphism could be in linkage disequilibrium with the actual gene variants related to the disease.

## Conclusions

Despite these limitations, the results of this study suggest that the RAGE pathway partially mediates AGE-induced MCI in diabetic individuals. The serum AGE-P level may serve as serum biomarker of MCI, and the sRAGE level is a predictor and even a potential intervention target of early cognitive decline in type 2 diabetes patients. Thus, the AGE-RAGE system may participate in the development of early cognitive decline in type 2 diabetes patients. Additional evidence, especially from longitudinal studies, is needed to elucidate whether the serum levels of AGE-P and RAGE could serve as predictive biomarkers of MCI in diabetic patients.

The interpretation of the data presented in this study exhibits certain limitations. First and most importantly, the present work is a case-control study, and a causal relationship between RAGE and cognitive function in T2DM could not be determined because data from magnetic resonance imaging or autopsy were unavailable. Thus, the relationship between brain pathology and diabetes mellitus could not be causally linked. Second, all of the recruited patients were hospitalized, and high rates of delirium and cognitive changes may be observed in a hospital environment. These factors may add some bias to our findings. Finally, the sample size was relatively small.

## Supporting Information

S1 File TREND (PDF)

S2 File Protocol of mild cognitive impairment in diabetes mellitus (DOC)
