# Supplementary material for: RAGE and AGEs in Mild Cognitive Impairment of Diabetic Patients: A Cross-Sectional Study
Source: PLoS One. 2016 Jan 8;11(1):e0145521. doi: 10.1371/journal.pone.0145521 (PMC4706319; doi:10.1371/journal.pone.0145521)
Supplement: S2 File — (DOC) [file pone.0145521.s002.doc]

**糖基化终产物致糖尿病认知障碍：BDNF-TrkB-CREB信号介导的海马重塑神经网络机制研究**

**Advanced glycation end products induced diabetic cognitive impairment: Neural network mechanisms of hippocampal BDNF-TrkB-CREB signal mediated remodeling**

**单位：东南大学附属医院内分泌科**

**资金来源：国家自然科学基金面上项目 SBE81370921**

**版本日期：2013年2月28日**

**项目负责人：王少华教授**

**Hospital: Affiliated Hospital of Southeast University, of Endocrinology**

**Funding: National Natural Science Foundation of China SBE81370921**

**Date: February 28, 2013**

本文件所包含的所有信息均应视为机密，同时东南大学附属中大医院对此保留独家所有权。这些信息的使用必须限于方案规定的目的，不论是何原因、以何形式，未事先得到东南大学附属中大医院内分泌科的书面同意，禁止披露、发表或与其他非授权人交流。

All the information contained in this document should be regarded as confidential, and affiliated zhongda hospitalof southeast university to retain sole ownership. The purpose of the use of these information must be limited to plan provisions, whatever the reason, in any form, did not obtain prior written consent of southeast university affiliated zhongda hospital endocrinology, prohibited to disclose, publish or communicate with any other unauthorized person.

**目 录 Contents**

**1.研究背景 Background**

**2.研究设计 Study Design**

**3.可行性分析 Feasibility Analysis**

**4.不良事件 Adverse events**

**5.严重不良事件 Serious adverse events**

**6.研究者速报事件 Researchers quick - reporting event**

**7.伦理和法规 Ethics and regulations**

**8.研究监查 Study Monitoring**

**9.管理细则 Management rules**

**1、研究背景 Background**

全球目前大约17.1亿糖尿病人，预计到2030年将会是36.6亿。中国糖尿病患病率高达11.6%，糖尿病前期患病率高达50.1%，约有1.57亿糖尿病人，而糖尿病前期约有6.9亿。大脑不仅极度依赖葡萄糖供能，而且同肾脏、心脏、视网膜一样，是糖尿病易累及器官。糖尿病可以造成一过性和永久性认知障碍。据临床学数据表明，糖尿病认知障碍患病率在糖尿病人群众为60%~70%。随着糖尿病发病率的增高、发病的年轻化，都预示着糖尿病相关并发症的增加，而糖尿病认知障碍的存在极大得损伤了患者的工作、学习、生活能力，给家庭和社会造成巨大负担。糖尿病可通过多种途径影响认知功能，包括高血糖、血脂异常、胰岛素缺乏、糖毒性及加速的Aβ沉积，胰岛素生长因子IGF-1缺乏，GSK3β基因缺陷，IDE，CETP, ABCA1，CLU，PS，CLUSTERIN，CYSTATIN基因突变及异常表达，但潜在的相关机制目前仍不清楚。

Currently, there are about 1.71 billion people with type 2 diabetes mellitus all over the world, it is expected to be 3.66 billion in 2030. The prevalence rate of diabetes in China is 11.6%, and the rate of pre-diabetes prevalence is 50.1%, there are about 157 million people with diabetes in China, and the pre-diabetes are about 690 million. Brain is highly dependent on glucose for energy, and it is the same as other organs such as kidneys, heart, retina which were easily impaired by diabetes. Diabetes can cause transient or permanent cognitive impairment. The clinical science data showed that the prevalence of diabetes cognitive impairment in people with diabetes was about 60% to 70%. The increasing incidence of diabetes, the younger age of diabetes, all indicated increased diabetes-related complications. At the same time, the presence of cognitive impairment can significantly influence the work, learning, life skills ability of the patients, which can cause a great burden of family and society. Diabetes can affect cognitive function through a variety of pathophysiology, including hyperglycemia, dyslipidemia, insulin deficiency, glucose toxicity and accelerated Aβ deposition, gene mutation of insulin-like growth factor (IGF-1), GSK3β, IDE, CETP, ABCA1, CLU, PS, CLUSTERIN, CYSTATIN, but the exact underlying mechanisms are still unclear.

然而遗憾的是，糖尿病认知障碍的发病机制迄今尚未能阐明。糖尿病与痴呆，特别是AD存在诸多共同的病理基础，高糖毒性、胰岛素及其信号传导异常、氧化应激及炎症反应均成为糖尿病认知障碍潜在的发病机制(Whitmer et al., 2007)。其中糖基化终末产物(Advanced glycation end products, AGEs)作为介导高糖毒性公认的生物效应分子，是在糖尿病高糖状态下葡萄糖与蛋白质上的游离氨基通过非酶反应产生的一组终末产物。

Though the pathogenesis of cognitive impairment in diabetes has not yet been able to be clarified. Diabetes and dementia, particular the Alzheimer`s disease, have many common pathological basis, such as the toxicity of high blood glucose level, abnormalities in insulin and its signaling, oxidative stress and inflammation. All of them could be potential pathogenesis of diabetic cognitive impairment (Whitmer et al., 2007). And the advanced glycation end products (AGEs), as recognized biological effector molecules mediates high glucose toxicity, was produced by non-enzymatic reaction with free amino groups under high glucose in diabetes mellitus.

研究表明糖尿病早期脑内葡萄糖水平即升高，遂通过与神经元的蛋白非酶糖基化反应，致AGEs 形成，由此促进糖尿病脑部相关慢性并发症发生，包括糖尿病性认知障碍(Mruthinti et al., 2006)及老化相关的病理改变(Mohamed et al., 1999)。

Studies have shown that increased brain glucose levels in the brain of diabetes mellitus, which can react with protein in neurons through non-enzymatic glycation reaction, causing the formation of AGEs, thereby promoting brain-related chronic complications of diabetes, including diabetic cognitive impairment (Mruthinti et al., 2006) and aging-related pathology (Mohamed et al., 1999).

既往研究认为AGEs致糖尿病认知障碍的主要机制为：①修饰tau 蛋白和β-淀粉样肽，参与AD 特征性病理形成；②其细胞毒性促神经元凋亡(Takeuchi et al., 2008; Byun et al., 2012)；③促进血管壁老化蛋白持续沉积(Messier et al., 2004)。AGEs的多种生物学效应是由晚期糖基化终产物受体（RAGE）实现的。

Past studies suggest that the main mechanisms of AGEs caused cognitive impairment are as follows, ① AGEs can cause the modification of tau protein and β-amyloid peptides which was involved in characteristic of AD pathology; ② AGEs can cause cytotoxic which further promote neuronal apoptosis (Takeuchi et al, 2008; Byun et al, 2012);. ③ promote sustained aging protein deposition in vascular wall (Messier et al, 2004).. A variety of biological effects of AGEs were mediated by advanced glycation end products receptor (RAGE).

人RAGE基因位于6p21.3，RAGE基因外显子Exon3密码子第82位碱基GGC→AGC突变，可引起功能性氨基酸甘氨酸向色氨酸转变（Gly-Ser）。已有研究证明Gly82Ser等位基因与心血管疾病，糖尿病并发症痴呆，尤其是阿尔茨海默病独立相关。

RAGE gene is located on 6p21.3 in human, the 82 codon base on RAGE exon Exon3 have GGC→AGC mutation, which can cause functional amino acid glycine to tryptophan change (Gly-Ser). It has been proved that Gly82Ser allele were independently accociated with cardiovascular disease, dementia, diabetic complications, particularly Alzheimer's disease.

细胞表面的全长RAGE可经溶蛋白性裂解失去与细胞膜结合部位，分泌入血。与此同时，人RAGE基因可因mRNA的选择性剪接而产生多种异形体，缺乏C端部分序列的异形体可与RAGE配体结合，并由细胞分泌出来形成内源性分泌性RAGE。以上两种方式可形成血清中可溶性RAGE（sRAGE）。sRAGE可与RAGE配体结合，阻断RAGE-配体反应，进而保护细胞免受损伤。目前研究发现sRAGE可作为RAGE的阻断剂，血清中RAGE水平已被证实与血管性并发症及认知障碍下降相关。

Full-length RAGE cell surface may lose binding site of the membrane by proteolytic cleavage, and were secreted into the blood. At the same time, RAGE have a variety of isoforms due to the alternative splicing of mRNA, and the isform which lack of a partial sequence of the C-terminal can combine with the ligands, it can be secreted and formed the endogenous secretory RAGE. These formed in two kinds of serum soluble RAGE (sRAGE). sRAGE binds to RAGE ligands and blocks RAGE- ligand reaction, thereby protects cells from damage. The previous study found sRAGE can function as a decoy of RAGE, and RAGE serum levels have been shown to be associated with decreased vascular complications and cognitive impairment decline.

动物实验却发现，显著认知障碍糖尿病鼠存在海马超微结构的变化而并未见明显毛细血管基底膜增厚、管腔狭窄等糖尿病血管的海马重塑可促进疾病状态下神经网络的形成和认知功能康复，这表明：（1）海马作为高级精神活动中枢，其神经元超微结构的破坏极可能成为糖尿病认知障碍的基本病理特征；（2）海马超微结构病变可能更多地源于高糖毒性这一糖尿病核心代谢因素的直接或间接效应，而非糖尿病血管病变所致。此外，海马还是成年哺乳动物脑内持续存在重塑的二个区域之一，新生与固有神经元建立突触联系参与完成脑内神经网络连接，成为海马损伤神经元修复促其发挥认知相关功能的结构基础。

Animal experiments have found a significant ultrastructure change of hippocampus in the diabetic mice with presence of cognitive impairment and no significant capillary basement membrane thickening, stenosis and other diabetic vascular remodeling in the hippocampus may promote the formation of disease states and neural networks cognitive rehabilitation, which showed that, (1) the hippocampus is a senior mental activity center, and the neuron ultrastructure damage in hippocampus is likely to become the basic pathology of cognitive impairment; (2) the ultrastructural lesions in the hippocampus may be caused by the toxicity of high glucose metabolic factors directly or indirectly instead of diabetic vascular disease. In addition, the hippocampus is one of the two persistence remodeling areas in the adult mammalian brain, the connection between newborn and intrinsic neuronal synaptic participated in brain neural network connections, become the structural basis of hippocampal neuronal damage repair related cognitive function.

BDNF主要由海马合成分泌且在脑内含量最高，生理条件下，BDNF促进成年海马NPCs增殖，新生神经元存活（ Sairanen et al.,2005）并保护受损神经元（Scharfman et al., 2006; Sairanen et al., 2007）。其高亲和力的转录则由转录调控子Ca 或cAMP 偶联的反应结合蛋白（ cAMP responsive-element bindingprotein，CREB）调控，BDNF使CREB 磷酸化而发挥其生物学活性(Fitzsimons et al.,2009)。：作为糖毒性效应分子，AGEs 致海马重塑障碍；BDNF-TrkB-CREB 通路作为其下游信号分子介导上述过程，阻碍了新生神经元生物学功能的发挥及其神经网络形成，成为糖尿病认知障碍的可能机制之一；BDNF(Val66Met)基因型可能与糖尿病认知障碍相关，并成为糖尿病患者伴认知障碍的候选易感基因之一。

BDNF is mainly synthesized and secreted by the hippocampus and the brain has the highest BDNF concentrations. In physiological conditions, BDNF can promote the proliferation of hippocampal NPCs in adults, survival of new neurons and protection of damaged neurons (Scharfman et al, 2006 (Sairanen et al, 2005.).; Sairanen et al., 2007). Its high affinity for transcription by transcription reaction regulator Ca or cAMP binding protein coupled (cAMP responsive-element bindingprotein, CREB) regulation, BDNF make CREB phosphorylation exert their biological activity (Fitzsimons et al., 2009). As the mediators of high blood glucose toxic effects, AGEs can induce the disorder of hippocampal remodeling; BDNF-TrkB-CREB pathway, as the downstream signaling molecules which mediate this process, hindering the biological function of new neurons and the formation of neural network, become one of the possible mechanisms of diabetic mild cognitive impairment; BDNF (Val66Met) genotype may be associated with cognitive impairment of diabetes mellitus, and become one of the candidate susceptibility genes associated with cognitive impairment in patients with diabetes.

本研究以AGEs对海马重塑及其神经网络影响为突破口，紧紧围绕AGEs-sRAGE轴及BDNF-TrkB-CREB信号通路及BDNF(Val66Met)基因多态性，发挥现已有跨学科交叉集成优势，多层面深入探讨AGEs致糖尿病认知障碍的海马重塑神经网络机制, 并联合运用多模态神经影像技术，在体水平进一步揭示其临床功能意义，以期最终发现糖尿病认知障碍的分子和影像学标记，对完善临床诊疗策略进行创建性的探索。本研究必将为探讨糖尿病状态下高级神经活动－情感障碍的分子机制提供新的思路和证据。

This study makes the remodeling and influence of AGEs on hippocampal neural network as a breakthrough, and focuses on AGEs-sRAGE axis, BDNF-TrkB-CREB signaling pathway and BDNF (Val66Met) gene polymorphism and make advantages of interdisciplinary integration benefits to investigate multidimensional depth AGEs cause of cognitive impairment in hippocampal neural network remodeling mechanisms and use multi-modal neuroimaging techniques, to reveal the levels of significance of clinical features, eventually discovery the molecular and imaging markers of cognitive impairment and to create sexual exploration on improving the clinical diagnosis and treatment strategies. This study will investigate the diabetic state of higher nervous activity - to provide new ideas and evidence of the molecular mechanisms of affective disorders.

**2、研究设计 Study design**

**2.1、研究目的 Objectives**

本研究以AGEs对海马重塑及其神经网络影响为突破口，紧紧围绕AGEs-sRAGE轴，RAGEGly82Ser基因多态性及BDNF-TrkB-CREB信号通路及BDNF(Val66Met)基因多态性，发挥现已有跨学科交叉集成优势，多层面深入探讨AGEs致糖尿病认知障碍的海马重塑神经网络机制, 并联合运用多模态神经影像技术，在体水平进一步揭示其临床功能意义，以期最终发现糖尿病认知障碍的分子和影像学标记，对完善临床诊疗策略进行创建性的探索。

This study makes the remodeling and influence of AGEs on hippocampal neural network as a breakthrough, and focuses on AGEs-sRAGE axis, BDNF-TrkB-CREB signaling pathway and BDNF (Val66Met) gene polymorphism and make advantages of interdisciplinary integration benefits to investigate multidimensional depth AGEs cause of cognitive impairment in hippocampal neural network remodeling mechanisms and use multi-modal neuroimaging techniques, to reveal the levels of significance of clinical features, eventually discovery the molecular and imaging markers of cognitive impairment and to create sexual exploration on improving the clinical diagnosis and treatment strategies.

**2.2、研究对象 Objects of study**

**2.2.1、入组标准： Inclusion criteria**

（1）糖尿病诊断符合WHO 1999年制订的标准，且明确诊断为2型。

(1) The diagnosis of diabetes was according to WHO 1999 criteria, and diagnosed as type 2 diabetes mellitus.

（2）年龄50～75岁。

(2) aged 50 to 75 years old.

（3）主诉记忆减退，并经他人证实。

(3) complained of memory loss, and confirmed by others.

（4）临床痴呆分级量表(Clinical dementia rating，CDR)0.5分。

(4) Clinical Dementia Rating Scale (Clinical dementia rating, CDR) 0.5 points.

（5）有足够的视觉和听觉分辨力接受神经心理学测试。

(5) Adequate visual and auditory resolution to accept neuropsychological testing.

（6）测试前12个月内CT或MRI头颅扫描无感染、梗死或其他局灶性损伤的证据，也无相关临床症状，但允许一个非关键性脑区域的腔隙性梗死且不被认为影响受试者的认知损害。

(6) No evidence of infection, infarction or other injury by head CT or MRI scans in 12 months before the test, and no clinical symptoms, but allow a non-critical area of the brain lacunar infarction which not be considered to influence the cognitive impairment of subjects.

（7）Hachinski缺血量表(Hachinski ischemia scale，HIS)得分小于或等于4分。

(7) Hachinski Ischemic Scale (Hachinski ischemia scale, HIS) score less than or equal to 4 points.

**2.2.2、排除标准：Exclusion criteria**

（1）近3个月内有糖尿病酮症酸中毒等急性并发症；严重心、肺、肾功能衰竭史；脑血管意外。

(1) Diabetic ketoacidosis and other acute complications within the past three months; severe heart, lung, kidney failure history; cerebrovascular accident.

（2）任何引起痴呆的神经系统疾病，包括AD、帕金森病、亨廷顿病、正常压力脑积水、脑肿瘤、进行性核上性麻痹、癫痫、慢性硬膜下血肿及多发性硬化，有严重头外伤史伴有持续神经功能缺损或已知的脑结构异常。

(2) Any neurological disease which can cause dementia, including AD, Parkinson's disease, Huntington's disease, normal pressure hydrocephalus, brain tumor, progressive supranuclear palsy, epilepsy, chronic subdural hematoma and multiple sclerosis, serious head trauma with persistent neurological deficits or structural abnormalities of the brain.

（3）既往2年内患抑郁症，汉密尔顿抑郁量表评分>10分。

(3)Suffering from depression in past 2 years, Hamilton depression scale score>10 points.

（4）其他可能导致脑功能紊乱的器质性和精神疾病。

(4) Other organic diseases or mental illness which possible cause brain dysfunction.

（5）筛选前1个月内使用过下列药物疗法：①中枢性B受体阻滞剂、麻醉药、甲基多巴和可乐定；②抗帕金森病药物，如筛选前2个月内使用过左旋多巴、金刚烷胺、溴麦角环肽、丙基麦角灵和司来吉兰；③神经镇静药和麻醉性镇痛剂；④苯二氮卓类(地西泮)和巴比妥类药物；⑤短效抗焦虑剂或镇静性药物的使用每周多于2次(注意筛选前72 h内不得使用镇静剂)；⑥激素；⑦有明显胆碱能或抗胆碱能不良反应的药物(如吡啶斯的明、三环抗抑郁药、氯苯甲嗪、奥昔布宁)；⑧抗癫痫药(苯妥英钠、苯巴比妥、卡马西平)；⑨华法林(苄丙酮香豆素)；⑩盐酸多奈哌齐、盐酸美金刚以及新近被认可的治疗AD的任何临床试验药物。

(5) a drug therapy used as followings months prior to screening: ①central B-blockers, drugs, methyldopa and clonidine; ②anti-Parkinson's disease drugs, such as filtering within two months ago used levodopa, amantadine, bromocriptine, propyl ergoline and selegiline; ③nerve sedative and narcotic analgesics; ④benzodiazepine class (diazepam) and Bobby duly drugs; ⑤use short-acting anxiolytic or sedative drugs more than twice a week (Note screening should not be used within 72 h before the sedatives); ⑥hormone; ⑦significant cholinergic or anticholinergic side effects drugs (such as pyridostigmine, tricyclic antidepressants, meclizine, oxybutynin); ⑧antiepileptic drugs (phenytoin, phenobarbital, carbamazepine); ⑨warfarin (benzyl warfarin); ⑩donepezil hydrochloride, memantine hydrochloride or any clinical trials of the drug recently approved to treat AD.

**2.3、神经心理量表测试和神经认知功能评估：**

**Neuropsychological tests and assessment of neurocognitive function**

简易智力状态量表( MMSE) 、蒙特利尔认知评估量表（MoCA）、连线测试A（Trail Making Test A）、连线测试B（Trail Making Test B）、听觉词语学习测验（Auditory Verbal Learning Test，AVLT）、言语流畅性测验（Verbal Fluency Test，VFT）、画钟测验（Clock Drawing Test，CDT）、数字广度测试（Digit Span Test，DST）、临床痴呆评定量表( Clinical dementia rating, CDR)、Hachinski缺血量表(HIS)、日常生活能力量表（Activity of daily living Scale, ADL）、宗氏抑郁自评量表（Self-rating Depression Scale，SDS）、汉密尔顿抑郁量表（17-Item Hamilton Depression Rating Scale ，HAM-D）

MMSE、MoCA、Trail Making Test A、Trail Making Test B、Auditory Verbal Learning Test，AVLT、Verbal Fluency Test，VFT、Clock Drawing Test，CDT、Digit Span Test，DST、Clinical dementia rating, CDR、HIS、Activity of daily living Scale, ADL、Self-rating Depression Scale，SDS、17-Item Hamilton Depression Rating Scale ，HAM-D

**2.4、临床资料采集： Clinical data collection**

大血管并发症方面：颈动脉斑块，冠心病病史，脑梗病史，下肢动脉血栓病史等。

微血管并发症方面：微量白蛋白尿，视网膜病变，周围神经病变等。

Macrovascular complications: carotid plaque, a history of coronary heart disease, cerebral infarction history, medical history of lower extremity arterial thrombosis.

Microvascular complications: microalbuminuria, retinopathy, peripheral neuropathy

血脂代谢：总胆固醇，低密度脂蛋白，高密度脂蛋白，甘油三酯，BMI，非酒精性脂肪肝等。

血糖：随机血糖，量表测量前后血糖，糖化血红蛋白，空腹血糖，C肽释放实验。

病史：糖尿病病程，高血压病史、病程，吸烟史、饮酒史、药物使用等。

Lipid metabolism: total cholesterol, LDL, HDL, triglycerides, BMI, non-alcoholic fatty liver.

Blood Glucose: random blood glucose, measured before and after the scale of blood glucose, glycated hemoglobin, fasting glucose, C-peptide release experiment.

History: duration of diabetes, hypertension, duration, smoking history, drinking history, drug use and so on.

**2.5、血标本收集与处理 Collection and processing of blood samples**

所有受试者均于入组次日晨8时测量血压后抽取患者空腹静脉血检查空腹血糖( fasting blood glucose, FBG) ，C肽，糖化血红蛋白(HbA1c) 、白蛋白及血脂全套，留取2mL静脉血，EDTA抗凝，半小时内4℃低温离心10000g×2min，血浆冻存于-80℃冰箱待测。

All subjects`s fasting blood were collected after the measurement of blood pressure to check fasting glucose (fasting blood glucose, FBG), C peptide, glycosylated hemoglobin (HbA1c), a full set of albumin and lipids, 2 mL blood were retained, EDTA anticoagulated, within half an hour at 4 ℃ centrifugation 10000g × 2min, plasma frozen at - 80 ℃ refrigerator tested.

**2.6、影像学检查 Imageological examination**

入组患者凡自愿行核磁功能检查者，行MRI平扫及fMRI数据收集，包括VBM技术检测全脑灰质体积、海马灰质体积以及密度；DTI和纤维示踪技术显示大脑白质纤维束；fMRI显示静息状态下海马等皮层功能区的激活状态。对激活皮质进行定位，比较激活广度和深度差异；

The group of patients who voluntarily NMR line function tests who underwent MRI scanning and fMRI data collection, including the detection of VBM whole brain gray matter volume, hippocampal gray matter volume and density; DTI and fiber tracing technique showed white matter fiber bundle; fMRI display static active hippocampus and other cortical functional areas under the state interest. To activate the cortex to locate, compare activation breadth and depth of differences;

**2.7、统计学分析：Statistical analysis:**

采用SPSS 19.0进行数据分析处理，计量资料以均数±标准差或中位数(M)表示。符合正态分布的计量资料采用独立样本t检验,非正态分布的计量资料采用非参数Mann-Whitney U 检验,定性数据分析采用卡方检验。两组间各项神经心理测试成绩差异大小的比较以标准化Z值表示(Z=认知障碍组的均数-正常组均数/正常组标准差)。相关分析采用Pearson检验。检验水准α = 0.05。

Data analysis was performed using SPSS 19.0, measurement data were presented as mean ± standard deviation or median (M) represented. Independent samples t test were used when the measurement data were normally distributed, non-normal distribution of measurement data were analysis by the non-parametric Mann-Whitney U test, qualitative data were analyzed using chi-square test. Comparative performance difference between the two groups of the size of neuropsychological tests to standardize Z value represents (Z = cognitive impairment group mean - mean normal / normal standard deviation). Pearson correlation analysis was performed using test. Significance level α = 0.05.

**2.9、技术路线 Study flowchart**

Type 2 diabetes mellitus（Met the inclusion and exclusion criteria）

Medical history collection after in-hospital, complete the relevant laboratory and imaging examination

Specimens from blood samples sent to the laboratory to separate the serum, blood cells

Collection of clinical data, and other information and perform the clinical scales

Serum levels of protein expression for ELSA Identification

Plasma is used to extract DNA, PCR

**、**

**3、可行性分析 Feasibility Analysis**

1. 本课题组组员认真学习并追踪了近年来发表的与本课题相关的重要专著和文献，把握糖尿病认知障碍临床和基础的最新研究进展，掌握了此领域研究的发展动态，熟练掌握课题涉及的各种技术，具备充分的可行性。

Our group members studied and tracked the recent publication of an important monograph and literature related to this subject seriously, and grasped the latest diabetes research progress in clinical and basic cognitive impairment, and mastered the developments in this field of research, mastering subjects involved various techniques and we have sufficient feasibility.

1. 课题组拥有一支踏实、严谨、高效的基础和临床科研队伍，梯队实力雄厚、结构合理，是圆满完成课题任务的组织保障。负责人王少华教授科研功底扎实，具备组织、管理、协调大型科研项目的能力。研究组主要成员均在临床和科研领域颇有建树。

（2）Our group has a practical, robust and efficient basic and clinical research team, echelon strength, reasonable structure, and it is the organizational guarantee of successful completion of the tas. Professor Wang Shaohua, head of research and a solid foundation, with the organization, management and coordination of large-scale research projects. Key members of the study group succeed in clinical and scientific fields.

1. 东南大学已建成包括学习科学中心、生物医学工程系、附属中大医院糖尿病研究所、脑血管病研究所、分子影像与功能影像实验室、基础医学院分子遗传学实验中心等脑科学和代谢疾病整体研究为特色的研究体系和机构，完全具备课题所需全部的相关技术支持和研究设备。

（3）Southeast university has built including learning sciences center, department of biomedical engineering, institute of diabetes, cerebrovascular disease research institute affiliated zhongda hospital, molecular imaging and functional imaging laboratory, basic medical molecular genetics experiment center brain science and metabolic diseases such as the overall research for the study of the characteristic of system and institution, include all the subjects required for all the related technical support and research equipment.

**4、不良事件 Adverse events**

**4.1、不良事件的判定 Determination of adverse events**

受试者在临床试验过程中（自签署知情同意书至最后1次随访期间）发生的任何不良的医疗事件，无论是否与试验药物有关，均定义为不良事件。

Subjects in the clinical trial process (from self-signed informed consent to the last follow-up) any adverse medical events, regardless of whether related to the test drug, were defined as adverse events.

**4.2、不良事件的处理 The processing of adverse events**

应如实填写不良事件记录表，记录不良事件的受试者、随访时间、编码用辞、研究分组、发生时间、严重程度、持续时间、与治疗的关系、采取的措施和转归。

Adverse events should be recorded in the sheet honestly, including subject of adverse events, follow-up time, with the speech coding, research groups, time of occurrence, severity, duration, relationship and treatment, and outcome measures.

**5、严重不良事件 Serious adverse events**

**5.1、严重不良事件的判定 Determination of serious adverse event**

严重不良事件是在临床试验过程中，使用任何剂量试验药物下出现的以下医学事件之一：导致死亡或危及生命（此处“危及生命”是指患者在发生严重不良事件时有死亡的危险，而不是假设某个严重不良事件更严重时就可能导致死亡）；需要住院治疗或延长住院时间；导致永久或严重的残疾/功能丧失；先天畸形或出生缺陷；有重要医学意义事件。

Serious adverse events mean during the clinical trials process, the occur of following events using the medical test drugs one at any dose: death or life-threatening (where "life-threatening" refers to a patient at a risk of serious adverse events of death, rather than assuming a more serious when serious adverse events may lead to death); the need for hospitalization or prolongation of existing hospitalization; cause permanent or severe disability / loss of function; congenital malformations or birth defects; there are important medical significance of the event.

**5.2、发生严重不良事件的处理 Process of serious adverse events**

任何严重不良事件，必须立即采取适当的治疗措施。

In case of any serious adverse events, appropriate measures must be taken immediately.

研究者应立即通知监查组代表。

Researchers should immediately notify the OMV group representatives.

研究者应在24小时内将已签名并注明日期的病例报告表中的相应页传真并电话通知至牵头单位东南大学附属中大医院王少华医生（电话：13390900488），由牵头单位向药监部门报告。应监查并记录该事件过程，获取相关的医疗记录和资料，注意保护受试者身份。

Researchers should be signed and dated case report form to the appropriate page within 24 hours fax and telephone notification to the Zhongda Hospital, Southeast University, Dr. Wang Shaohua lead unit (Tel: 13390900488), the lead unit to the pharmaceutical sector reports. It should be monitored and recorded the event procedure, access to relevant medical records and information, to protect the identity of the subject.

首次报告后，研究者应在获知更新的信息后（如果有）1个工作日内向监查组代表报告相关信息。

After the first report, researchers should be informed of updated information (if any) in a work day and report to the relevant information to OMV Group Representative's.

**5.3、发生严重不良事件的随访 The follow-up of serious adverse events**

发生严重不良事件，必须对受试者随访，直到临床完全恢复及实验室检查结果恢复正常，或直到病情稳定。这可能意味着随访要持续到受试者结束临床研究后，而且监查组可能要求进行附加的研究。随访信息必须记录在严重不良事件随访表中并交给相关的监查员。

Once serious adverse events occur, subject must be followed-up, fully restored until clinical and laboratory test results returned to normal, or until a stable condition. This may mean follow-up to continue after the end of clinical research into the subject, and OMV group may require additional research. Follow-up information must be recorded in a serious adverse event follow-up table and handled to the relevant inspectors.

如果在研究药物停用后任何时候有引起研究者注意的严重不良事件，而且研究者认为有合理的可能性该严重不良事件是由研究药物引起的，就应该向监查组报告。

If there is a serious adverse event caused by the researchers noted that the study drug after the disabled, and researchers believe there is a reasonable likelihood that serious adverse events are caused by the study drug, it should be reported to the audit group.

**6、研究者速报事件 Researchers rapid reporting event**

药物过量 Overdose drug

研究药物过量定义为：临床试验中任何大于日推荐/计划或处方剂量两倍的剂量。无论是意外或有意、是否有症状、严重或非严重，都应加急报告给申办者（1个工作日内）。

Study drug overdose defined as: clinical trials more than the recommended daily any / program or twice the dose prescribed dose. Whether accidental or intentional, whether symptomatic, severe or serious, should be expedited report to the sponsor (one working day).

**7、伦理和法规 Ethics and laws**

**7.1、伦理原则 Ethical principles**

临床研究将按照第18届世界医学大会（赫尔辛基，1964）制定的原则，及世

界医学大会制定的所有适用修订案，及ICH关于药物临床研究管理规范的指南实行。

The clinical study will follow the principle made by the 18th World Medical Assembly (Helsinki, 1964) and all applicable amendments Medicine Conference profession developed, and clinical research on drugs ICH guidelines for the implementation of management practices.

**7.2、法律和法规 Laws and regulations**

临床研究的实行将遵照所有国际性的法律法规和实施临床所在的国家级法律法规（中国），及任何适用的指南。

The clinical study will be implemented in compliance with all international laws and regulations and implementing national law where the clinicalLaws and regulations (China), and any applicable guidelines.

**7.3、知情同意 Informed consent**

受试者入选本研究前，研究者或研究者指派的人员应尽可能用受试者或其法

定代理人能理解的语言和/或术语，充分告知本研究的目的、程序、可能的风险及获益。受试者参与临床研究前，应签署书面的知情同意书，由受试者或受试者

的法定代理人，及进行知情同意讨论的人员亲笔签名和署上日期并存档。受试者

可保留知情同意书副本。

Subjects enrolled in the study before the designated investigator or researcher should as far as possible with the subject or method

Language and / or terms are an agent can be appreciated, fully informed of the study purpose, procedures, possible risks and benefits. Preclinical studies subjects participated, should sign a written informed consent form, the subject or the subject

Legal representative, and an informed consent discussion with signatures and dates on the Department and archived. Subjects

You may retain a copy of informed consent.

**7.4、机构审查委员会/独立伦理委员会Institutional Review Board / Independent Ethics Committee（IRB/IEC）**

研究者或牵头单位必须将临床研究方案提交给适当的伦理委员会（IRB/IEC），并且要求把伦理委员会主席签名和注明日期的伦理委员会（IRB/IEC）的书面批准/赞同意见送交一份复印件给牵头单位。

Lead researcher or clinical research program unit must submitted research protocol to the appropriate ethics committee (IRB/IEC), and requires the written approval of the Chairman of the Ethics Committee Ethics Committee signed and dated (IRB / IEC) is / agree with a submitted copies to the lead unit.

临床研究（研究编号，临床研究方案题目及版本编号），审核的文件（临床研究方案，知情同意书，研究者手册，研究者简历等），投票人员名单及其资格，及审核日期应在书面（IRB/IEC）批准/赞同意见中清楚说明。牵头单位在收到签有日期的书面批准/赞同意见的复印件前，不会向研究中心发出研究产品，临床研究也不会开始。临床研究期间，任何对临床研究方案的修正或调整均应递交给伦理委员会（IRB/IEC）。任何可能影响患者安全或影响临床研究继续进行的事件，均应通知伦理委员会，尤其是任何关于安全性的变化。所有研究者手册的更新资料也应送至伦理委员会（IRB/IEC）。如果有要求，应每年递交伦理委员会（IRB/IEC）一份进展报告，临床研究结束时，送交一份临床研究结果的总结。

Clinical studies (study number, title and clinical research programs version number), the audit file (the clinical study protocol, informed consent, the investigator's brochure, the researchers resume, etc.), a list of personnel and their qualifications to vote, and the review date should be written (IRB / IEC) approval / endorsement observations clearly. Focal signed before the date of receipt of the written approval / agree with copies, not send to the Research Center research products, clinical studies will not start. During clinical studies, amend or adjust any of the clinical research program shall be submitted to the Ethics Committee (IRB / IEC). Anything that might affect patient safety or clinical research event to proceed, shall inform the ethics committee, especially any change on security. All updates investigator's brochure should also be sent to the Ethics Committee (IRB / IEC). If requested, the Ethics Committee should submit annually (IRB / IEC) a progress report at the end of clinical research, delivered a summary of the clinical findings.

**8、研究监查 Study monitoring**

**8.1、研究者的职责 Researchers responsibilities**

研究者保证实施临床研究时会遵守临床研究方案、ICH关于药物临床研究管理规范的指南，及任何适用的法规要求。

Researchers will ensure compliance with the clinical study protocol implementing clinical studies, ICH clinical research on management practices guidelines, and any applicable regulatory requirements.

要求研究者确保遵守临床研究方案要求的所有程序，及由牵头单位提供的所有研究程序（包括安全规章）。研究者同意根据牵头单位提供的指令，提供可靠的临床研究方案要求的数据和所有资料，以准确清晰易读的方式牵头单位代表有权直接使用原始文件。

Researchers ensure compliance with all program requirements, and all the research programs (including safety regulations) provided by the focal clinical research program requirements. Researchers agree that the lead unit in accordance with instructions provided by the clinical research program to provide reliable data and all the information required to accurately legible manner on behalf of the lead units have the right to direct the use of the original file.

研究者可任命其它人员作为从属研究者，在遵守临床研究方案的条件下协助实施临床研究。所有从属研究者均应及时任命并列出名单。从属研究者由研究者监督，并且向研究者负责。研究者将向其提供临床研究方案的复印件及所有必要资料。

Researchers may appoint another person as a slave investigator to assist in the implementation of clinical research in compliance with the conditions for clinical research programs. Researchers shall promptly appoint all subordinate lists and lists. Dependent researchers supervised by the investigator, and the investigator in charge. Researchers will be provided with all necessary information and a copy of the clinical research program.

**8.2、原始文件的要求 The requirements of the original file**

根据ICH关于药物临床研究管理规范的指南，监查组必须对照原始文件检查CRF的记录，除了预先定义的直接记录在eCRF上的原始数据。知情同意书要包含一份声明，声明患者同意牵头单位正式授权的人员、伦理委员会（IRB/IEC）及法规部门有权直接使用支持病例报告表数据（如，患者的医疗档案、预约工作簿、原始实验室记录等）的原始资料。此类人员，由职业保密性所限，必须为所有个人身份及私人医疗信息保密（根据保密章程）。

According to ICH clinical studies on the drug management practices guide, OMV group must file checking against the original CRF records, the original data on the eCRF addition to direct recording predefined. Informed consent should contain a statement that the patient agrees focal duly authorized officer, the Ethics Committee (IRB / IEC) and the regulatory authorities the right to directly support the case report form data (such as the patient's medical records, appointment workbook, original laboratory records, etc.) to the original. Such persons, by the Occupational confidentiality constraints, must all personally identifiable information confidential and private health (under confidentiality charter).

**8.4、数据管理 Data Management**

数据全部录入由统计分析人员按要求独立完成统计分析，再由主要研究者写出研究报告。

All data are required to be analysis by the statistical staffs independently, then write research reports by the principal investigator.

**9、管理细则 Management rules**

**9.1、****履历 Curriculum vitae**

临床研究开始前，将每位研究者和副研究者履历的复印件提供给牵头单位，

履历内容限于经验、资格和培训。

Preclinical studies begin, copies each investigator and deputy investigator resume to the lead unit, Curriculum vitae content limited experience, qualifications and training.

**9.2、研究中心记录的保存 Preservation of Center records**

研究者必须维护所有研究文件的机密性，并且采取措施防止这些文件意外或过早受到毁坏。

Researchers must maintain the confidentiality of all research papers, and take measures to prevent these files being destroyed by accidental or premature.

研究者在临床研究完成或中止后，将这些研究文件保存至少五（5）年。

研究者在临床研究完成或中止后销毁任何研究的基本文件前，必须通知牵头单位。如果研究者的个人状况使得他/她不能确保档案的保存，研究者应告知牵头单位，并且相关记录将会转移至双方均同意的授权者。

Researchers in the clinical studies completed or aborted, to save these research papers at least five (5) years.

Former investigator in the clinical studies completed or aborted destroy any study of the basic documents, must notify the lead unit. If the personal situation of researchers so that he / she can not ensure that you save the file, the researcher should inform the lead unit, and related records will be transferred to both parties agree authority.
